# On the Influence of Capillary-Based Structural Health Monitoring on Fatigue Crack Initiation and Propagation in Straight Lugs

**DOI:** 10.3390/ma12182965

**Published:** 2019-09-12

**Authors:** Marc Moonens, Eric Wyart, Dieter De Baere, Michaël Hinderdael, Julien Ertveldt, Zoé Jardon, Galid Arroud, Patrick Guillaume

**Affiliations:** 1 Mechanical Engineering Department, Vrije Universiteit Brussel, Pleinlaan 2, 1050 Brussels, Belgium; Dieter.De.Baere@vub.be (D.D.B.); Michael.Hinderdael@vub.be (M.H.); Julien.Ertveldt@vub.be (J.E.); Zoe.Jardon@vub.be (Z.J.); Galid.Arroud@vub.be (G.A.); Patrick.Guillaume@vub.be (P.G.); 2 Cenaero ASBL, Rue des frères Wright 29, 6041 Gosselies, Belgium; eric.wyart@cenaero.be

**Keywords:** capillary-based structural health monitoring, fatigue crack growth, fatigue crack initiation, XFEM, straight lug

## Abstract

This paper addresses the influence on the fatigue life induced by the implementation of a capillary-based structural health monitoring methodology, patented under the name eSHM. It consists in integrating structurally small and pressurized capillaries into the component, so that when a fatigue crack breaches the capillary network, it results in a leak flow to the open atmosphere and loss of pressure in the galleries which is detected by a pressure sensor. The novelty of the proposed system resides in the opportunity to locate the capillary according to the designer’s need, as one resorts to additive manufacturing for the part production. However, the presence of these galleries in highly stressed regions raises concerns about crack initiation at the capillary itself and accelerated fatigue crack growth. This paper aims at the quantification of the influence the eSHM has on the fatigue behavior of the component and the determination whether this influence is significant or not. To that purpose, numerical simulations on a straight lug component, using the finite elements and eXtended Finite Elements Methods (XFEM), are performed. Various capillary sizes and shapes are assessed, so as to enable a general conclusion on the impact of the eSHM methodology in straight lugs.

## 1. Introduction

The history of aviation is sadly enough quite abundant with examples of propagating fatigue cracks that lead to a structural failure. Actually, the firsts phenomenological understandings of the fatigue phenomenon in metals came after the series of crashes experienced by the first jetliner to enter commercial service, namely the Comet I aircraft [1,2,3]. In the time when it was designed, the deterioration of mechanical properties due to the presence of cracks was largely underestimated. Nowadays, even if our knowledge concerning the fatigue of materials has dramatically progressed, fatigue failures continue to happen and in almost all sectors of the industry. An insight of this can be given through the review of failure causes in aircraft components done by Findlay and Harrisson in 2002 [4,5], which reveals that fatigue is the dominant failure mode in aeronautics (55% of the failures), well ahead of corrosion (16% of failures) and overload (14%; the rest being wear and stress/temperature corrosion). This illustrates very well that due to the complexity of the fatigue phenomenon in metals, design offices are still struggling with issues related to fatigue. Therefore, in the author’s view, this calls for a new paradigm in the approach followed to deal with fatigue cracks. One of the innovative approaches that could be followed in that context and which is thought to be very promising is known under the generic terminology of “Structural Health Monitoring”.

Formally, Structural Health Monitoring (SHM) can be defined as the process of acquiring and analyzing the data from on-board sensors to evaluate the health of a structure [6]. Up to now, multiple SHM systems have been developed, with different target applications. Most of these systems used to be vibrations based [7,8]. However, structural health monitoring systems have been a thriving research domain for several years now, implying the emergence of innovative approaches for performing SHM. It would be impossible to be complete here, but technologies such as carbon nanotubes based structural health monitoring or comparative vacuum monitoring sensors illustrate very well this diversity [9,10,11]. In all cases, robustness remains a crucial attention point in the design of structural health monitoring systems [7,12].

**Figure 1:**
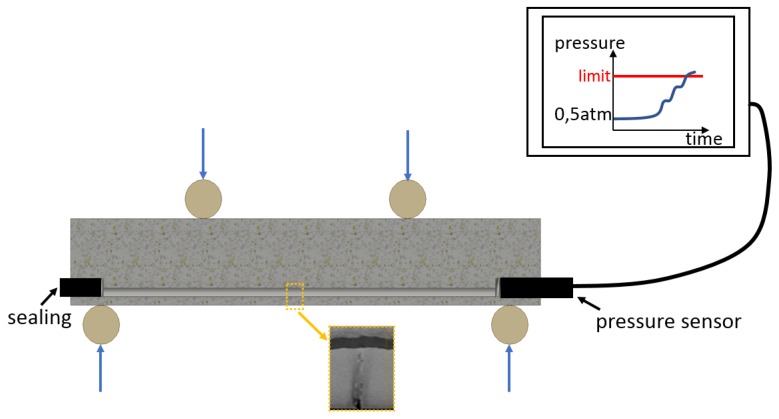
Illustration of the eSHM working principle on a four point bending test sample.

Aside from this, the advent of additive manufacturing opens the door to the development of innovative concepts, such as smart metals. Therefore, the authors of this work have been active on an alternative implementation of the SHM principle where the geometric freedom provided by additive manufacturing could be exploited. The previously conducted research efforts resulted in a technology that is now patented (patent number EP2801809A1, [13]) under the name “effective Structural Health Monitoring (eSHM)”. The system consists in integrating over- or under-pressurized capillaries (the capillaries are thus internal channels) into the to-be-monitored component, so that when a fatigue crack breaches the capillary network, a leak flow is created, and the pressure equilibration between the capillary and the open atmosphere is detected by a pressure sensor. Figure 1 illustrates the working principle on a four point bending test sample equipped with an under-pressurized capillary. The pressure is initially set to 0.5 atm in the capillary before the experiment starts, but as soon as the propagating fatigue crack reaches it (clearly seen on the micro-XCT image), air from the open atmosphere enters the capillary, and internal capillary pressure rises sharply. This pressure rise is detected by a sensor, which triggers an alarm when a pre-defined value has been reached, thus revealing the presence of the crack [14]. In that sense, a parallel can be drawn between the proposed structural health monitoring approach and the previously developed Helicopter Blade Crack Detection System (patent number US5205710A, [15]). However, the proposed methodology is on purpose mainly targeted at additively manufactured components [6]. The geometric freedom offered by additive manufacturing implies that the capillaries can be placed according to the designer’s needs. In other words, the proposed SHM methodology enables close and robust monitoring of the regions which were identified by design studies as prone to crack initiation. For illustration, it can be mentioned that it would be impossible to monitor the entire lug hole region of the lug studied below should it be manufactured with conventional methods. Moreover, the proposed technology requires no significant signal processing and electronic hardware, as only the capillary pressure should be measured and monitored (refer to the experimental investigations in [16]). Detailed information regarding the working principle of this novel SHM methodology can be found in the patent [13].

While additive manufacturing has shown significant potential for widespread usage in production of complex and/or customized parts, this potential has been dampened by, notably, the variation of mechanical properties, which includes the fatigue behavior of these parts [17]. Indeed, it is well-known in the literature that printing parameters such as technology used (powder bed fusion, laser metal deposition, etc.), scanning speed, powder flow rate (non-exhaustive list) have notably a strong influence on the microstructure and residual stresses present in the component, and hence, on the mechanical properties. Moreover, post-treatments applied (if any) on the part also result in a mechanical behavior different compared to the as-built conditions. As a matter of illustration, it has been reported that the Hot Isostatic Pressing process (hipping) contributes to a significant improvement of fatigue properties, and possibly to properties that are similar to their conventional counterparts [18,19,20,21]. The dispersal of mechanical properties of AM parts inevitably reduces their reliability and thus impinges the adoption of additive manufacturing in engineering applications [17]. In that context, it is thought that besides offering high level of robustness, the proposed SHM philosophy could also contribute in revealing the full potential of additive manufacturing.

However, the introduction of capillaries in regions which are potentially subject to high stresses might raise concerns on the influence the eSHM could have on the fatigue life of the structure. Günther et al. [22] have shown that test samples with internal channels have reduced fatigue lives compared to samples without internal channels, the reduction being dependent on the topology of the channels. However, still according to Günther et al., this is primarily due to rapid crack initiation at the primary roughness of the internal channels, and secondly to the modified topology of the specimens. In the present research, the effect of capillary roughness on the reduction of fatigue life has not been taken into account. Indeed, technologies such as hybrid manufacturing (combination of additive and subtractive manufacturing) or chemical etching could potentially enable a drastic reduction in capillary roughness levels in the near future [23,24,25,26]. Moreover, in the context of the eSHM system, the roughness influence has already been experimentally investigated (see [27]). Therefore, one focuses here on the consequences of the presence and topology of virtually smooth capillaries on fatigue life. This is particularly important for the further development of the system, as the eSHM system has to offer a quick detection of growing fatigue cracks (by being placed “as close as possible” to the most probable initiation site), while not affecting the component’s function (the capillary should not trigger initiation nor reduce fatigue life). As a matter of fact, this study can be seen as the completion of our initial work [14], as both initiation and propagation are addressed, and as additional capillary topologies and initial flaws are studied. This should enable to draw a more general conclusion on the eSHM influence on fatigue behavior of equipped lugs.

## 2. Crack Initiation Considerations

### 2.1. Numerical Framework

**Figure 2:**
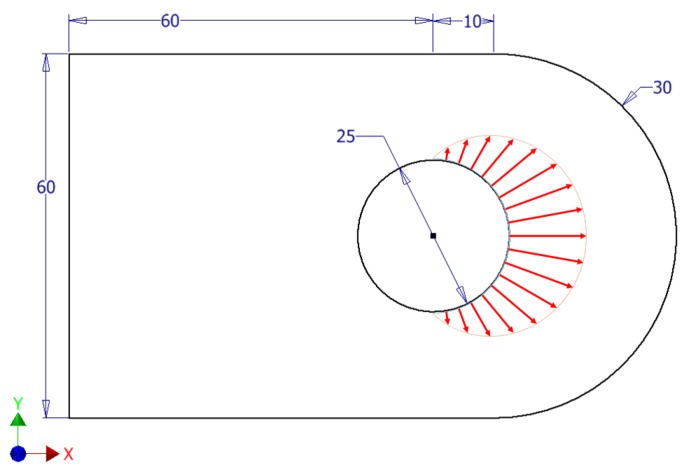
Dimensions (in mm) of the lug modeled, and pin loading modeled as an equivalent pressure with cosine distribution around the hole.

The idea behind this research is to study the impact of the implementation of the eSHM technology in a potential practical application. Since the eSHM is originally targeted at aerospace applications, it has been decided to work on straight lug components. Indeed, this type of connecting element is frequently used in aeronautics, and is also very prone to fatigue failures (fretting and/or corrosion are very likely to initiate a crack) [28]. Amongst the abundant literature on lugs, it was decided to work on the configuration studied by Schijve in [29], where fatigue crack growth experiments have been performed on various aluminum straight lugs (in-plane geometry depicted in Figure 2, thickness of 5 mm) subject to a cyclically varying axial loading. The maximal pin force is $F_{max}=21$ kN and the load ratio is R=0.33. It must be noted that this force is modeled through this entire work as an equivalent pressure having a cosinusoidal distribution on the pin hole surface. Moreover, even though a lug with integrated capillary would be barely impossible to manufacture without resorting to additive manufacturing, the material properties that are used in the present work are similar to the properties of the Al2024-T3 material used in the experimental work done in [29]. These are: a Young’s modulus of 73,000 MPa, a Poisson’s ratio of 0.33, and a yield stress of 368 MPa. It is thus assumed that the material is isotropic, and that the variations of the mechanical properties due to the printing process do not influence the results of the comparison provided the same material properties are enforced in all the models.

**Figure 3:**
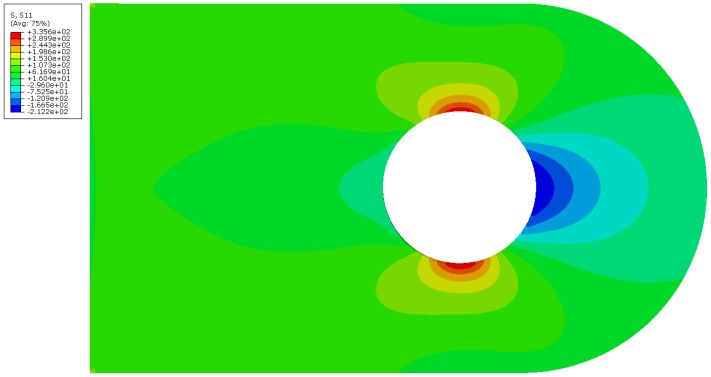
$S_{11}$ field on the lug, highlighting the most probable region for initiation under cyclic and purely axial loading.

**Figure 4:**
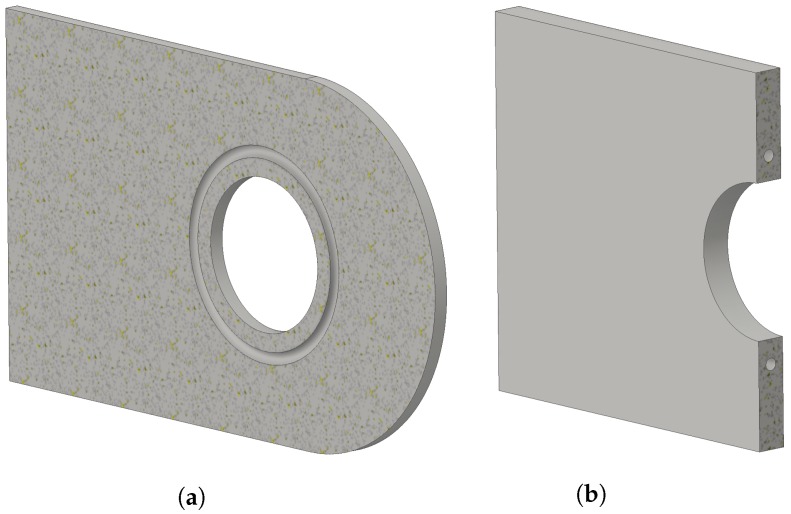
Cut views of the lug equipped with eSHM on a XY plane at mid-thickness (**a**) and on a YZ plane centered at the lug hole (**b**). The capillary around the hole is clearly visible (in the shown topology, the capillary is circular with a diameter of 2 mm).

Figure 3 shows the axial stress field $\sigma_{xx}$ (the *x*-axis is along the longitudinal direction, as shown on Figure 2), further noted as $S_{11}$, on the lug when subject to the maximal axial pin loading. The highest tensile stresses are found on the top and bottom regions of the pin hole surface and reach 336 MPa (effectively corresponding to a stress concentration factor of 2.8 with respect to the maximum net tension stress, see [29]). Excluding manufacturing defects, the highest probability of crack initiation is thus also found there. These two regions of high tensile stresses will hence further be referred as the “initiation regions” and are the focus in this part of the study. It must be noted that, considering this stress distribution, capillaries should be placed and limited to the neighborhood of these initiation regions. However, in an actual practical application, the loading could have a non-axial component. Moreover, in testings, notches are introduced in these area’s in order to trigger initiation at a known place, which is of course not done in an operational component. As a consequence, the initiation region is never as clearly defined as what has been assumed here. Therefore, capillaries are integrated all around the hole of the studied lug, so as to enable detection of any crack that initiated at the hole surface (see Figure 4).

While the eSHM system has the advantage of a very simple principle of operation, conferring a significant robustness to the system, and since the parts equipped with eSHM have to be manufactured by additive manufacturing, it still presents a considerable design freedom in terms of engineering. Indeed, the capillary shape is in principle arbitrary and can be “chosen” by the designer, as well as its dimensions. However, keeping in mind that one should avoid salient edges and convex shapes, this design freedom is restrained and one will limit oneself to capillaries of circular and elliptical cross section. Nevertheless, the diameter/aspect ratio of the capillary can be tuned, and this part of the study aims at quantifying in which proportion the capillary diameter/aspect ratio influences the crack initiation behavior. Besides, the “edge-to-edge” distance between the bottom of the capillary and the lug hole surface, referred here as distance “a” (in accordance with the terminology used in the works [26,30,27]), is also a parameter in the engineering of the crack detection system. A smaller edge-to-edge distance would imply a quicker detection of the propagating crack, and again, one aims at quantifying the influence of this design parameter on the crack initiation. It must be noted that the pressure inside the capillary also is another design parameter, but it has been shown in a previous study that, at the pressurization levels used, no effect was observable on the stress field in the component [14].

**Figure 5:**
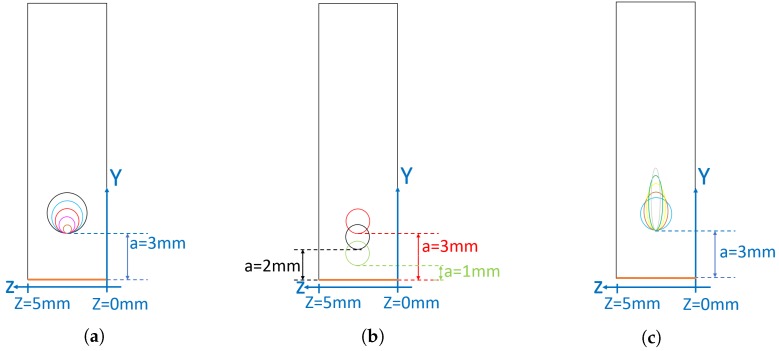
Schematics of the upper cross section of the lug when looking in a YZ plane centered at the lug hole (one can refer to Figure 4b), illustrating the different implementations of the eSHM (capillaries) that have been assessed. In particular, different capillary diameters (**a**), edge-to-edge distance (**b**) and aspect ratio’s (**c**).

The parametric study led in this work therefore concentrates on the influence of capillary shape (circular or elliptical), dimension (diameter and aspect ratio), and edge-to-edge distance with respect to the monitored region (lug hole surface). Figure 5 illustrates the different configurations under consideration. Static simulations of the different configurations subject to the maximum pin loading ($F_{max}=21,000$ N) have been run, and the resulting maximum tensile stresses on the lug hole surface and capillary surface are compared to the situation of a “standard” or “reference” lug where no capillary has been integrated. For the sake of accuracy of the numerical results, one has taken advantage of the symmetry of the problem and only a quarter of the lug has been modeled. The mesh, consisting of about 700k (exact number depending on the eSHM configuration) linear hexahedron elements, has been obtained after several refinement steps to ensure the use of a converged mesh.

### 2.2. Circular Capillary—Influence of Diameter

**Figure 6:**
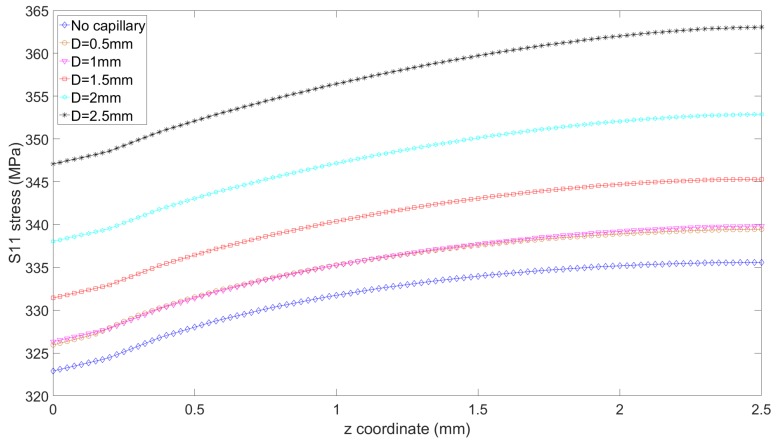
Axial stress $S_{11}$ on the most critical line of the pin hole surface (bottom line in Figure 5, highlighted in orange), across the lug thickness, and as a function of the capillary diameter.

In this section, capillaries of circular cross section are considered. The influence of different diameter sizes on the stress field in the component is studied. In particular, as here above mentioned, one focuses on the axial stress in the initiation region ($S_{11,ini}$) and on the capillary surface, as these are considered to be the best indicators of the influence the eSHM has on the initiation behavior. These results have been retrieved for five different diameters, namely 0.5 mm, 1 mm, 1.5 mm, 2 mm and 2.5 mm (respectively shown in brown, mauve, red, light blue and black on Figure 5a and on Figure 6), all being located so that the edge-to-edge distance “a” remains equal to 3 mm. These diameters corresponds to realistic values of what can be or will be achieved in a near future with the current or forthcoming additive manufacturing technologies. Indeed, technologies based on Powder Bed Fusion (PBF) have proven to be able to print capillaries down to 1 mm in diameter size, while technologies such as Direct Energy Deposition (DED) are currently limited to 2 mm. However, considering the evolution of the metal printing capabilities, and the advent of hybrid manufacturing, it is credible to think that smaller capillaries will be soon achievable. Therefore, it felt relevant to model capillaries as small as 0.5 mm in diameter, even if those are not yet printable.

The axial stress values in the initiation region (cross-section bottom line in Figure 5) can be seen in Figure 6. As symmetry is used in the model, the results are presented for z=0 mm to z=2.5 mm, while the lug thickness is actually 5 mm. The reference lug experiences a maximum tensile axial stress of 336 MPa in the initiation region (also seen on Figure 3). On the graph of Figure 6, it can be seen that the presence of the capillary inside the lug raises the tensile axial stress level in that region, and therefore reduces the crack initiation life. However, it can be noticed that capillaries of very small dimensions (0.5 and 1 mm) have a very limited impact on the maximum stress level (339 MPa instead of 336 MPa). Table 1 summarizes this data, and also presents the maximum axial tensile stress on the capillary surface. It is noteworthy to realize that in all cases, the maximum stress on the capillary surface remains significantly inferior to the stress in the initiation region. This implies that in the case of a smooth capillary, a crack would normally not initiate at the capillary itself, as the lug hole surface remains the region where the amplitude of tensile stresses variations is the highest.

### 2.3. Circular Capillary—Influence of Edge-to-Edge Distance

Bringing the capillary closer to the initiation region (thus, reducing the distance “a”) is desirable for earlier detection of fatigue cracks that would develop at the lug hole surface. However, the influence it has on stress raise in the initiation region and at the capillary surface has to be studied. In the experimental investigations around the eSHM, the distance “a” is traditionally set to 2 mm [26,27]. Therefore, for the purpose of this parametric study, the diameter of the capillary was kept constant at 1.5 mm, and edge-to-edge distances equal to 3 mm, 2 mm and 1 mm have been considered (respectively shown in red, black and green on Figure 5b).

The results are presented in Table 2. These demonstrate that earlier crack detection (smaller “a” distance) inherently comes with a raised stress level in the initiation region, but this raising remains very limited (increase of 1.9% between a=3 mm and a=1 mm). Moreover, even for an edge-to-edge distance of 1 mm, the tensile stress level on the capillary surface remains inferior to the tensile stress level in the initiation region. Again, this implies that the lug hole surface would remain the initiation site. It is however noteworthy to realize that in practice, due to the current limitations in additive manufacturing technologies, the roughness level in the capillary acts as a significant stress raiser, and implies that when brought too close to the surface, cracks actually initiate at the capillary instead of at the surface. This has been thoroughly studied in the work by Günther et al. [22]. This is also the reason why experimental testing around the eSHM has traditionally used an edge-to-edge distance of 2 mm [26,27]. From this, it can be inferred that capillary roughness is the main challenge to the practical implementation of the eSHM, as the presence of the capillary itself should not affect the crack initiation site nor significantly the crack initiation life, provided the capillaries are small with respect to the lug dimensions.

### 2.4. Elliptical Capillary—Influence of Ellipse Aspect Ratio

Finally, it has to be assessed whether there would be an added-value to using capillaries of elliptical cross section instead of circular cross section. Indeed, these would definitely be more complex to manufacture, but it is deemed that this could have a beneficial impact on the tensile stress level in the initiation region and more significantly on the capillary surface. To that purpose, capillaries of various aspect ratios, ranging from 1 (circular capillary) to 4, but of identical cross sectional area (1 mm${}^{2}$) and constant edge-to-edge distance are considered. These configurations are shown in Figure 5c.

The results of the simulations performed on these lugs is shown in Table 3. It can be seen that the impact on the stress level in the initiation region is negligible. However, as it is expected, the use of an elliptical cross section affects quite significantly the stress level at the capillary surface, as the maximum value of tensile stress attained on the capillary surface drops from 226 MPa (circular capillary) to 174 MPa (ellipse of aspect ratio 4), thus resulting in a 23% decrease on the surface. As a consequence, it indicates that the use of an elliptical cross section could enable to position the capillary closer to the lug hole surface (hence achieving faster detection) while minimizing the risk of initiation at the capillary itself.

## 3. Crack Propagation Considerations

### 3.1. Numerical Framework

When a crack grows from the initiation region, due to the reduction of cross-sectional area in the plane where the crack propagates, it can be expected that the propagation speeds will be higher than in the reference lug, at least when the crack propagates through the region where the capillary is located. Hence, the objective of this section is to quantify the repercussions of the acceleration on the crack growth life. This quantification is done in function of capillary shape and size. To that purpose, fatigue crack growth simulations have been run on lugs equipped with different configurations of the eSHM, namely those of Figure 5a,c. The results are then compared to computations run on the reference lug (in which there is no capillary). Two types of initial defect are considered: a part-through crack of initial length $a_{0}=1$ mm (to be consistent with the study of Schijve [29]), and a quarter-elliptical crack of initial lengths $a_{0}=1$ mm and $b_{0}=1$ mm, both located at the initiation region (top of the lug hole surface). Part-trough cracks and quarter-elliptical cracks are actually the most fore-coming type of defects in aeronautical lugs [30].

The objective here is to make a comparison between a reference lug and lugs equipped with the eSHM, so that to be able to infer up to what extend the capillaries do affect the crack growth life. The crack propagation model used may thus remain simple, and therefore, the Paris law has been referred to [31,32], considering a Paris exponent n =3.58 and a Paris coefficient of $C=1.3651e-13\frac{mm}{cycle}\cdot {\left(MPa\sqrt{mm}\right)}^{-n}$. The fracture toughness of aluminum 2024-T3 is $K_{c}=$1010 MPa$\sqrt{mm}$, so that the lugs are considered to have failed when the cracks reach a length of $a_{f}=$12 mm (the maximum stress intensity factors (mode I) on the crack front reach $K_{c}$ for that crack length).

**Figure 7:**
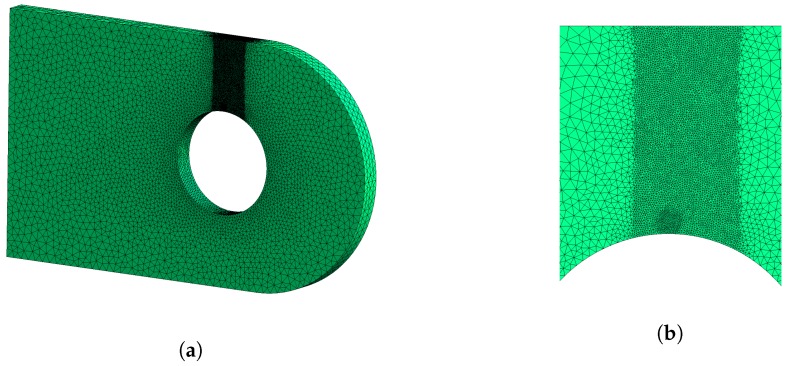
Mesh used in the fatigue crack propagation simulations. (**a**): overview. (**b**): highly refined region around the crack.

All the fatigue crack growth simulations have been performed with Morfeo, developed by Cenaero [33,34]. In the software, the propagation is driven by a user defined crack propagation step Δa. The software then computes the corresponding ΔN, number of cycles required to propagate the crack by Δa. One should note that Δa has to be set to a value ensuring that the propagation path is properly computed, and that the time integration yielding the ΔN is correctly evaluated. Since the software is based on the XFEM method, the crack position in the mesh is spotted by level sets, which are at each step updated based on propagation length and direction [33,34,35]. All the meshes used in this section have been generated using the open source pre- and post-processor “Gmsh”, developed by Geuzaine and Remacle [36]. They were obtained after several refinement steps, ensuring the use of a converged mesh. The mesh was highly refined in the crack region, and particularly in the initial defect region (see Figure 7), to ensure for converged stress intensity factors on the complete crack path. Depending on the configuration, the mesh totalizes between 850 k and 930 k linear tetrahedron elements.

### 3.2. Influence of Capillary Diameter on Crack Growth Life

**Figure 8:**
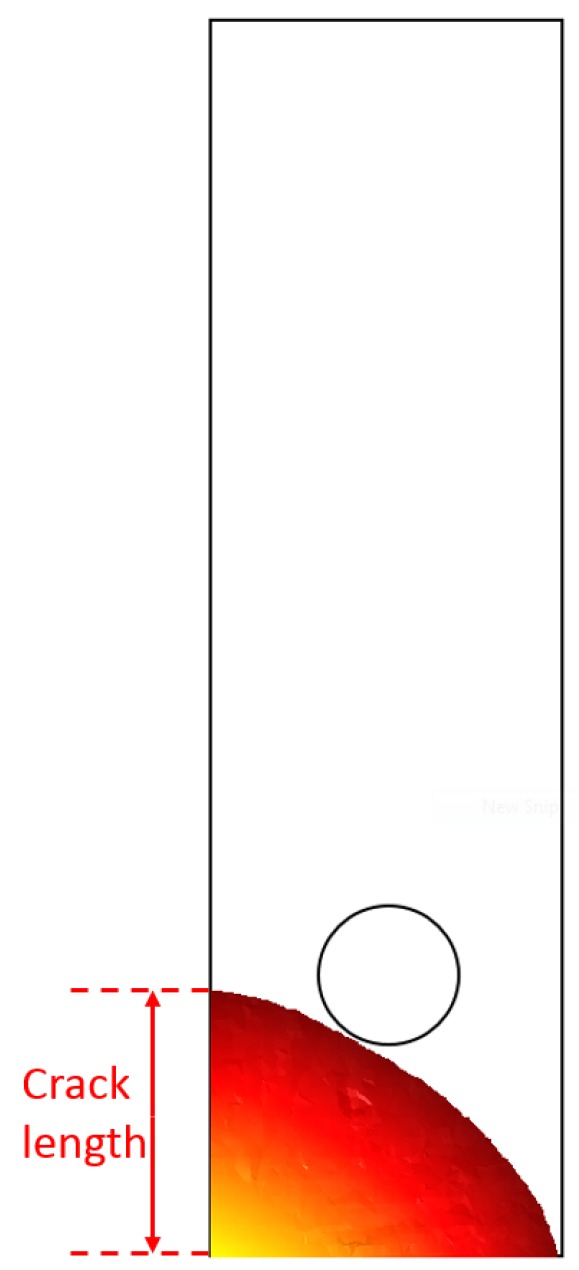
Crack front just before reaching the capillary (2 mm diameter) after propagation of the initial quarter-elliptical defect.

In what follows, the “crack length” refers to the distance between the lug hole surface and the intersection between the crack front and the side surface of the lug. This choice has almost no influence on the crack growth curve of the part-through crack since the crack front curvature is very limited. However, concerning the quarter-elliptical crack, this definition implies that the number of cycles to detection (corresponding to the moment where the crack breaches the capillary) does not correspond to a crack length equal to 3 mm (see Figure 8).

**Figure 9:**
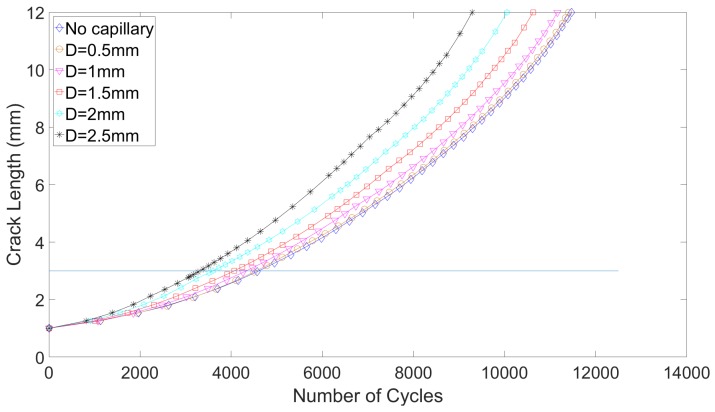
Fatigue crack growth curves for various capillary diameters, for a part-through crack.

**Figure 10:**
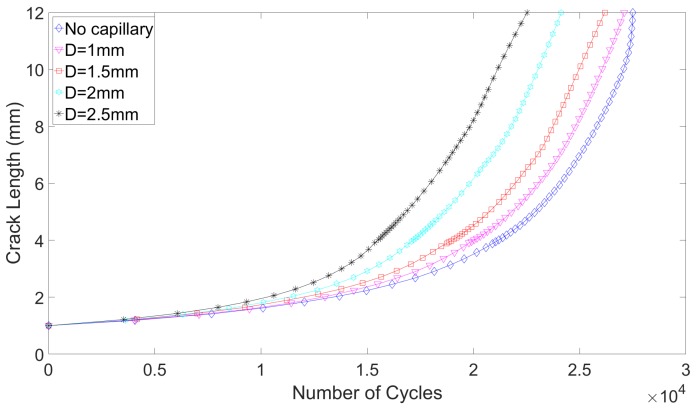
Fatigue crack growth curves for various capillary diameters, for a for a quarter-elliptical crack.

The crack growth curves are presented in Figure 9 and Figure 10. The number of cycles to failure and number of cycles to detection are given respectively in Table 4 and Table 5. It is interesting to note that even though a simple crack propagation model has been used, the fatigue life obtained for the reference lug and for the part-through crack, namely 11,470 cycles, agrees fairly with the experimental works of Schijve, where the fatigue life of the specimens oscillated between 13,500 and 15,500 cycles [29]. For the quarter-elliptical initial defect, the numerical results cannot really be compared to the experimental works of Schijve, as the initial defect introduced in the experiment (a corner crack) is different than the one modeled in the present research.

Several interesting observations can be made from these crack propagation computations. First, for both types of defects, small capillaries have a limited negative impact in terms of the fatigue crack growth lifes. The impact that would be tolerated by a component manufacturer would inevitably be application dependent. However, to set ideas, one considers here that a reduction by 5% of the crack growth life compared to a reference lug remains acceptable. With this assumption, capillaries of 0.5 mm and 1 mm are acceptable designs. Conversely, for large capillaries, the severe stress state on the propagation plane implies that the stress intensity factor field on the crack front is also more severe. In the first propagation step computed, the maximum mode I stress intensity factor on the reference lug for a part-through crack is 586 MPa$\sqrt{mm}$, while it already reaches 644 MPa$\sqrt{mm}$ for the lug equipped with a 2.5 mm diameter capillary. The propagation speeds are thus already larger, even before the crack reaches the capillary. This is clearly seen on Figure 9. Second, it is worth noting that this trend is consistent with the effect capillaries have in terms of crack initiation. This illustrates the interest of working with relatively small capillaries (compared to the dimensions of the component), which is also the direction aimed for in the research around the eSHM methodology. Third, for the part-through crack, the detection occurs early in the crack propagation life, and can be further improved, should it be needed, by bringing the capillary closer to the initiation region. Indeed, as shown in Table 4 and in Figure 9, the remaining post-detection crack growth life represents 60% of the total crack propagation life ($N_{f}$). However, due to the lower propagation rates in the early stage (clearly seen in Figure 10), this is not true anymore for the quarter-elliptical crack. Indeed, the Remaining Useful Lifetime after detection is reduced in this case to barely 30% of $N_{f}$. Therefore, alternative designs, such as integrating two ex-centered capillaries, could be envisaged to remedy this when needed. Finally, it must be noted that the presence of the capillary has no influence on the crack path, which is in all cases straight in the *y*-direction.

### 3.3. Influence of Capillary Shape on the Crack Growth Life

**Figure 11:**
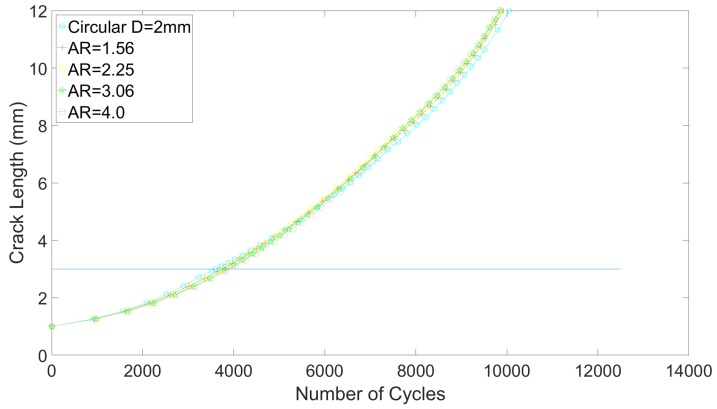
Fatigue crack growth curves for various capillary aspect ratio and in the case of part-through crack.

The crack propagation curves obtained for a part-through crack are shown in Figure 11. It can be clearly seen that varying the aspect ratio of the capillary actually has a negligible effect, as the impact on the crack propagation life remains below 2.5% variation (see Table 6). Increasing the aspect ratio of the capillary implies that the local reduction in area felt by the crack is less important than for circular capillaries. Crack propagation speeds in the neighborhood of the capillary are hence lower for elliptical capillaries. However, due to the vertical extension (along the *y*-axis) of the elliptical capillary, the time frame during which the crack propagates in a reduced available sectional area is also longer. This counterbalances the positive effect of lower propagation speeds, hence explaining why small difference in crack propagation life is obtained.

## 4. Conclusions

In the present work, the influence on fatigue life of the implementation of the effective Structural Health Monitoring (eSHM) methodology has been dealt within the context of straight lugs.

It has been shown that the inclusion of small capillaries (0.5 or 1 mm diameter) has a negligible influence on both crack initiation behavior and on fatigue crack propagation life. Conversely, large capillaries have a significant negative impact on crack growth life and crack initiation. This brings to the conclusion that proper application of the effective Structural Health Monitoring methodology requires extra engineering work at the design stage of the component, as a bad implementation can result in degraded fatigue performances. Moreover, this also sheds light on the importance of further research on hybrid manufacturing in the frame of the effective Structural Health Monitoring methodology, since that technology is deemed to enable the inclusion of capillaries that remain smooth while being very small in size.

It was also shown that the different configurations studied here present the drawback of late detection for quarter-elliptical cracks. However, thanks to the additive manufacturing process, many other configurations can be thought of to remedy this, should it be a problem for a particular application. One could think of integrating two side capillaries instead of one centered capillary, or use the weight spared with the inclusion of the capillary to increase the thickness of the lug when tolerances permit. In other words, the optimization space available in the frame of the configuration and implementation of the eSHM is very wide, and only a portion of that space has been explored here. This is especially true as additional features can be foreseen in the scope of the eSHM, such as crack localization and load monitoring. The scope of possibilities offered by the eSHM is very wide, and further research efforts should be devoted to the development of the eSHM methodology.

In this work, one important challenge to the possible practical utilization of the eSHM has been overcome. Several other hurdles exist before certification could be envisaged, but this work has demonstrated the interest and potential of equipping engineering components with the effective Structural Health Monitoring methodology. In particular, the authors intend to build a demonstrator lug equipped with this novel structural health monitoring technology, following the design recommendations of the present work.

## Figures and Tables

**Table 1:** Maximum axial tensile stress values in the initiation region ($S_{11,ini}$) and on the capillary surface ($S_{max,cap}$) in function of capillary diameter (D). The results are compared with the reference lug, in which no capillary is integrated.

D (mm)	$S_{11,ini}$ (MPa)	$\frac{S_{11,ini}}{ref}$	$S_{max,cap}$ (MPa)
Reference	336	-	-
0.5	339	+0.9%	198
1	340	+1.2%	204
1.5	345	+2.6%	214
2	352	+4.8%	226
2.5	363	+8.0%	245

**Table 2:** Maximum axial tensile stress values in the initiation region ($S_{11,ini}$) and on the capillary surface ($S_{max,cap}$) in function of edge-to-edge distance (a).

a (mm)	$S_{11,ini}$ (MPa)	$\frac{S_{11,ini}}{ref}$	$S_{max,cap}$ (MPa)
Reference	336	-	-
1	351	+4.5%	260
2	347	+3.3%	226
3	345	+2.7%	214

**Table 3:** Maximum axial tensile stress values in function of capillary cross section aspect ratio (AR).

AR	$S_{11,ini}$ (MPa)	$\frac{S_{11,ini}}{ref}$	$S_{max,cap}$ (MPa)
Reference	336	-	-
1 (circular)	352	+4.8%	226
1.56	350	+4.2%	199
2.25	349	+3.9%	183
3.06	348	+3.6%	176
4.0	347	+3.3%	174

**Table 4:** Fatigue crack growth lives ($N_{f})$ and cycles to detection ($N_{d}$) as a function of capillary diameter, in the case of part-through crack. The variation of fatigue life compared to the reference lug (Δ) is also given, as well as the Remaining Useful Lifetime after detection ($RUL_{d}$).

D (mm)	$N_{f}$	Δ	$N_{d}$	${RUL}_{d}$ (% of $N_{f}$)
Reference	11,470	-	-	-
0.5	11,400	−0.6%	4500	61
1	11,160	−2.7%	4470	60
1.5	10,630	−7.3%	4070	62
2	10,050	−12.4%	3605	64
2.5	9290	−19%	3380	63

**Table 5:** Fatigue crack growth lives ($N_{f})$, variation with respect to the reference lug (Δ), cycles to detection ($N_{d}$) and post-detection Remaining Useful Lifetime ($RUL_{d}$) as a function of capillary diameter and in the case of quarter-elliptical crack.

D (mm)	$N_{f}$	Δ	$N_{d}$	${RUL}_{d}$ (% of $N_{f}$)
Reference	27,490	-	-	-
0.5	27,220	−1%	19,970	26
1	27,080	−1.5%	19,100	29
1.5	26,180	−4.8%	18,100	31
2	24,130	−12.2%	16,870	30
2.5	22,520	−18%	15,070	33

**Table 6:** Fatigue crack growth lives ($N_{f}$), variations with respect to the circular capillary case ($\Delta_{circular}$) and cycles to detection ($N_{d}$) as a function of capillary aspect ratio, in the case of part-through crack.

AR (-)	$N_{f}$	$\Delta_{\mathrm{circular}}$	$N_{d}$
1	10,050	-	3605
1.56	9920	−1.3%	3805
2.25	9860	−1.9%	3900
3.06	9860	−1.9%	3990
4	9820	−2.3%%	4030
